# To Correspondents

**Published:** 1857-06

**Authors:** 


To Correspondents.—We have on hand several letters and short articles which shall receive due attention in our next No.

